# How common is alcohol use disorder in posttraumatic stress disorder? A systematic review, meta‐analysis, and moderator analysis

**DOI:** 10.1111/ajad.70200

**Published:** 2026-09-16

**Authors:** Maria Olivia Pozzolo Pedro, Manoela Pozzolo Pedro, Gabriella Zimmer Monteiro, Valquíria Custodio Klaumann, Priscila Dib Gonçalves, Silvia S. Martins, João Maurício Castaldelli‐Maia

**Affiliations:** ^1^ Department and Institute of Psychiatry University of São Paulo São Paulo Brazil; ^2^ Institute of Psychiatry Federal University of Rio de Janeiro Rio de Janeiro Brazil; ^3^ Heidelberg Hospital Curitiba Brazil; ^4^ Secretary of Health of Paraná Curitiba Brazil; ^5^ Department of Psychiatry Columbia University Irving Medical Center New York New York USA; ^6^ Department of Epidemiology Mailman School of Public Health, Columbia University New York New York USA; ^7^ Perdizes Institute (IPER) University of São Paulo São Paulo Brazil

## Abstract

**Background and Objectives:**

Comorbidity between posttraumatic stress disorder (PTSD) and alcohol use disorder (AUD) complicates care, yet reported prevalence varies widely across settings and methods. We estimated AUD prevalence among adults with PTSD and identified sources of variability.

**Methods:**

We searched PubMed, Google Scholar, the Virtual Health Library, and SciELO through June 2025, without language restrictions. All included studies were published in English. Observational studies reporting current or lifetime AUD in adults with PTSD were included (20 studies; 185,432 participants). Two reviewers independently appraised quality. Multilevel random‐effects meta‐analysis, trim‐and‐fill, sensitivity analyses, and meta‐regressions were conducted.

**Results:**

After excluding studies that relied solely on screening instruments, current AUD studies were insufficient for meta‐analysis (*k* = 3); results are restricted to lifetime prevalence. Pooled lifetime AUD prevalence was 35.58% (95% confidence interval [CI]: 24.48%–48.48%), with high heterogeneity (*I* ^2^ = 99.37%). Trim‐and‐fill identified no missing studies. Meta‐regression indicated that clinical sample source (vs*.* community), inpatient care setting (vs*.* outpatient), and veteran status (vs*.* non‐veteran) were associated with higher lifetime AUD prevalence; a higher proportion of female participants was associated with lower prevalence, and a current versus lifetime PTSD assessment with higher prevalence.

**Discussion and Conclusions:**

This review identifies sample characteristics that moderate AUD burden within PTSD populations: clinical settings, inpatient care, and veteran status predict higher prevalence, whereas greater female representation predicts lower rates. These findings support population‐specific screening and integrated, trauma‐informed care.

**Scientific Significance:**

This is the first meta‐analysis quantifying clinical and demographic moderators of AUD in PTSD samples, informing risk‐stratified screening and population‐tailored treatment allocation.

## INTRODUCTION

Posttraumatic stress disorder (PTSD) and alcohol use disorder (AUD; a diagnostic spectrum ranging from mild to severe under diagnostic and statistical manual [DSM]‐5 criteria^1^) represent two of the most clinically consequential psychiatric conditions in the general population.^2^ Although up to 70% of individuals across 24 countries may encounter traumatic stress at some point in their lives, only approximately 4% develop PTSD following such exposure,^3^ with prevalence ranging from roughly 6% (lifetime) in nationally representative U.S. samples^4^ to a point prevalence of approximately 26.5% in countries with recent war histories.^5^ Globally, the estimated 12‐month prevalence of AUD is approximately 8.6% among men and 1.7% among women.^6^ Notably, while AUD has historically been more prevalent among men, national longitudinal data indicate a progressive narrowing of this sex gap, with rising prevalence among women across multiple high‐income countries in recent decades.^7^, ^8^ When these disorders co‐occur, the clinical burden is amplified: individuals with comorbid PTSD and AUD experience poorer psychosocial functioning and substantially more complex treatment trajectories than those with either condition alone,^9^ as well as higher rates of suicidal ideation and increased mortality.^10^

The strong co‐occurrence of PTSD and AUD is supported by a well‐established theoretical and empirical framework. The self‐medication hypothesis proposes that individuals with PTSD use alcohol to temporarily alleviate distressing symptoms such as hyperarousal, intrusive memories, and insomnia.^11^ The affective processing model of negative reinforcement elaborates on this process, proposing that repeated alcohol use becomes entrenched through its capacity to attenuate negative affective states, thereby maintaining and potentially exacerbating PTSD symptoms over time.^12^ Beyond behavioral mechanisms, shared neurobiological substrates, including overlapping stress response systems (e.g., hypothalamic‐pituitary‐adrenal axis) and common reward pathways (e.g., ventral tegmental area), provide a biological foundation for this comorbidity.^13^ Furthermore, large‐scale genome‐wide association studies have demonstrated that PTSD shares a positive genetic liability with problematic alcohol use, but not with general alcohol consumption, underscoring the specificity of this genetic overlap.^14^

Empirically, prior reviews have documented that the prevalence of alcohol misuse among individuals with PTSD varies widely, ranging from approximately 10%–61% across studies employing different assessment timeframes.^15^ This substantial variation is unlikely to reflect true differences in comorbidity rates alone; rather, it likely reflects heterogeneity in sample characteristics, including clinical setting, demographic composition, and population type (e.g., veterans vs*.* civilians, inpatient vs*.* community samples). While these reviews have established the existence of this comorbidity,^15^ none has systematically examined how clinical and demographic moderators shape prevalence estimates specifically within PTSD‐defined samples using meta‐regression. Identifying and quantifying these moderators is essential for translating pooled estimates into actionable, population‐specific clinical guidance across the range of PTSD diagnostic approaches represented in the literature.

Veteran populations face multiple compounding risk factors, including elevated occupational trauma exposure and high rates of comorbid traumatic brain injury and chronic pain, that may confer substantially elevated risk for AUD compared to civilian samples.^16^, ^17^, ^18^ Beyond veteran status, both care setting and sample source are expected moderators of AUD prevalence: individuals presenting to clinical or inpatient settings characteristically exhibit greater symptom severity and comorbidity burden than those identified through community surveys, a well‐documented selection effect attributable to treatment‐seeking biases whereby more severely affected individuals are disproportionately represented in specialized healthcare settings.^19^ Finally, sex composition warrants formal moderation testing: although epidemiological data consistently document higher AUD rates among men,^7^, ^8^ the progressive narrowing of this gap and the underrepresentation of women in veteran and clinical samples introduce heterogeneity across studies that cannot be assumed away. Veteran status, care setting, clinical versus community sample source, and sex composition therefore constitute the primary candidate moderators evaluated in the present review.

In contrast to the predominant focus in the literature on detecting PTSD among individuals in treatment for AUD,^15^ this systematic review shifts the analytical lens to examine the prevalence of AUD specifically among individuals already diagnosed with PTSD. This PTSD‐focused approach reflects a clinically relevant assessment sequence: in settings where PTSD has already been identified, such as trauma‐specialized outpatient programs, veterans' services, and psychiatric inpatient units, quantifying the burden of comorbid AUD is essential for guiding screening intensity and treatment planning. Critically, this approach does not imply a causal or temporal directionality between the two conditions; rather, it addresses a practical clinical question about comorbidity burden within an established PTSD population. The present review therefore systematically estimates AUD prevalence within PTSD‐defined samples and employs meta‐regression to identify the clinical and demographic moderators most relevant to population‐specific screening and treatment allocation.

## METHODS

### Protocol and registration

This systematic review was conducted in accordance with the PRISMA (Preferred Reporting Items for Systematic Reviews and Meta‐Analyses) guidelines for transparent reporting (PRISMA 2020 Checklist—Supplementary Material [File S5]) and the MOOSE (Meta‐analysis of Observational Studies in Epidemiology) guidelines for meta‐analyses of observational studies. This review is part of a broader project investigating the prevalence of AUD among individuals with depressive disorders, anxiety disorders, and PTSD. The protocol was prospectively registered on the Open Science Framework (OSF) with the following identifier: (DOI 10.17605/OSF.IO/9UAMB).

### Search strategy and study selection criteria

A systematic search was conducted in PubMed, Google Scholar, Virtual Health Library (BVS) and SciELo for studies published up to June 30, 2025. The search strategy combined terms related to AUD, PTSD, and epidemiological studies, without language or date restrictions, though all were ultimately in English (Table S1). Reference lists of included studies and relevant reviews were manually screened to identify additional eligible articles.

Studies were included if they met the following criteria: (1) observational design (cross‐sectional or cohort); (2) adult population (≥18 years) with a primary diagnosis of PTSD; (3) reported prevalence or incidence estimates of comorbid AUD; (4) used standardized diagnostic criteria based on the Diagnostic and Statistical Manual of Mental Disorders (DSM) or the International Classification of Diseases (ICD), operationalized through structured clinical interviews or validated diagnostic instruments capable of generating a confirmed AUD diagnosis, such as the SCID, CIDI, MINI, or AUDADIS; studies relying solely on screening tools (e.g., AUDIT, CAGE) that yield probable rather than confirmed diagnoses were excluded; and (5) provided sufficient data to calculate proportions and 95% confidence intervals (CIs). Studies focusing on non‐human samples, qualitative data, or with overlapping samples were excluded. Interventional studies, including clinical trials, were excluded because their eligibility criteria often screen out individuals with active AUD, which would distort naturalistic prevalence estimates. Observational studies were retained because they capture AUD as it presents in defined PTSD populations; non‐clinical population‐based samples remained eligible only when AUD was established with a validated diagnostic instrument rather than a screening tool. For the purposes of this review, AUD was operationally defined to encompass AUD as per DSM‐5, as well as its predecessor diagnoses, alcohol abuse and alcohol dependence under DSM‐III/III‐R/IV, reflecting the nosological transition across the study period.

In instances of database redundancy, a hierarchical selection process was employed to retain a single representative study per cohort, such as number of individuals. Priority was given to the publication reporting the largest sample size or the most comprehensive diagnostic breakdown specific to the primary outcome (total comorbidity). Furthermore, secondary analyses restricted to specific temporal sequences (e.g., incidence of AUD only following PTSD onset) were excluded to ensure estimates reflected the total burden of diagnostic comorbidity regardless of onset order.

### Data extraction and quality assessment

Data were extracted using a standardized form. Extracted variables included: first author, year of publication, country, sample type (community or clinical), total sample size, number of participants with a confirmed PTSD diagnosis (i.e., the denominator for AUD prevalence calculations, which may differ from total sample size in population‐based designs), percentage of female participants, mean age, diagnostic criteria, and AUD and PTSD assessment timeframe (current vs*.* lifetime) disagreements were resolved by consensus.

Methodological quality was assessed using an adapted version of the Joanna Briggs Institute (JBI) critical appraisal checklist for prevalence studies. This tool evaluates risk of bias across nine domains encompassing sample frame adequacy, recruitment strategy, sample size justification, subject description, data coverage, diagnostic validity, measurement reliability, statistical appropriateness, and response rate. Any discrepancies in scoring were resolved through consensus or consultation with a third reviewer.

### Statistical analysis

To account for varying data structures across diagnostic timeframes, different meta‐analytical approaches were employed. For lifetime AUD prevalence, a multilevel random‐effects meta‐analysis was conducted to account for the statistical dependency between effect sizes derived from the same database (e.g., distinct populations such as elderly, veterans, and specific geographic groups nested within the same data source). This employed a three‐level structure: individual effect sizes (Level 1) nested within studies (Level 2), further nested within their data sources (Level 3), such as distinct waves of the National Epidemiologic Survey on Alcohol and Related Conditions (NESARC), from which three non‐overlapping subgroup analyses were derived. In this multilevel approach, proportions were stabilized using the logit transformation to ensure mathematical compatibility with the three‐level model structure.

Pooled prevalence estimates and their corresponding 95% CIs were calculated using a restricted maximum‐likelihood (REML) estimator to quantify between‐study heterogeneity (*τ* ^2^). Statistical heterogeneity was assessed using Cochran's *Q* test (with significance set at *p* < .10), Higgins and Thompson's *I* ^2^ statistic, and *τ* ^2^. For the multilevel models, *I* ^2^ was decomposed to indicate the proportion of total variance attributable to between‐study (Level 2) and between‐cluster (Level 3) heterogeneity. *I* ^2^ values of 25%, 50%, and 75% were considered low, moderate, and high heterogeneity, respectively.

To evaluate the robustness of the findings, leave‐one‐out sensitivity analyses were performed by iteratively removing one study at a time and recalculating the pooled prevalence and heterogeneity indices. Potential publication bias was assessed through visual inspection of funnel plots and Egger's regression test adapted for multilevel structures where appropriate. A *p*‐value below 0.05 was considered indicative of potential bias. When asymmetry was detected, Duval and Tweedie's trim‐and‐fill method was applied to estimate the number of potentially missing studies and compute adjusted pooled estimates, using a standard random‐effects model as per methodological constraints for this specific technique.

Moderator analyses were conducted to investigate sources of heterogeneity. Multilevel mixed‐effects meta‐regression models were applied to lifetime AUD data. These moderators were selected a priori based on established theoretical and empirical rationales: sex reflects known demographic gradients in AUD epidemiology; sample source and care setting serve as proxies for illness severity and sampling context; veteran status captures a population with documented elevated risk for comorbid PTSD and AUD; geographic region and diagnostic framework address variation introduced by cultural context and nosological differences; and decade of publication indexes secular trends in both diagnostic practice and AUD prevalence. Studies employing a 12‐month AUD assessment window were incorporated into the lifetime pool; AUD assessment timeframe (lifetime/anytime vs*.* 12‐month) was therefore included as a moderator to formally test whether this classification introduced systematic differences in prevalence estimates. The timeframe of the PTSD diagnosis (lifetime vs*.* current) was similarly included, as studies requiring an active PTSD syndrome at assessment may recruit systematically more severe clinical profiles than those accepting a lifetime diagnosis.

For the moderator analysis of female proportion, a multilevel mixed‐effects model was consistently used, as the exclusion of studies with missing data resulted in a subset that retained the nested structure. Results were reported as regression coefficients with corresponding 95% CIs and *p*‐values, and *R* ^2^ was used to indicate the proportion of heterogeneity explained by moderators. All statistical analyses were conducted using R version 4.3.2 with the meta and metafor packages.

## RESULTS

### Study selection and characteristics

The literature search initially retrieved 2248 records; deduplication reduced this number to 1562 unique citations (Figure 1). Following title/abstract screening and full‐text assessment, 16 articles retrieved through the database search and 4 additional articles identified via manual searching fulfilled the inclusion criteria. Across the 20 studies, a total of 185,432 participants were enrolled. Sex‐specific data were available for 15 studies, comprising 162,121 males and 20,400 females; the remaining five studies (*n* = 2911) did not report participants' sex (Table 1). The evidence base consisted of seven cohort investigations and 13 cross‐sectional studies. All studies reported prevalence estimates. Regarding alcohol‐related exposures, four studies assessed alcohol dependence, four examined alcohol abuse, and 14 assessed AUD. Pre‐DSM‐5 diagnoses of alcohol dependence and alcohol abuse were operationally treated as AUD‐spectrum indicators throughout this review. Studies were published across four decades, with one study from the 1980s, five from the 1990s, one from the 2000s, 11 from the 2010s, and two from the 2020s.

**Figure 1:**
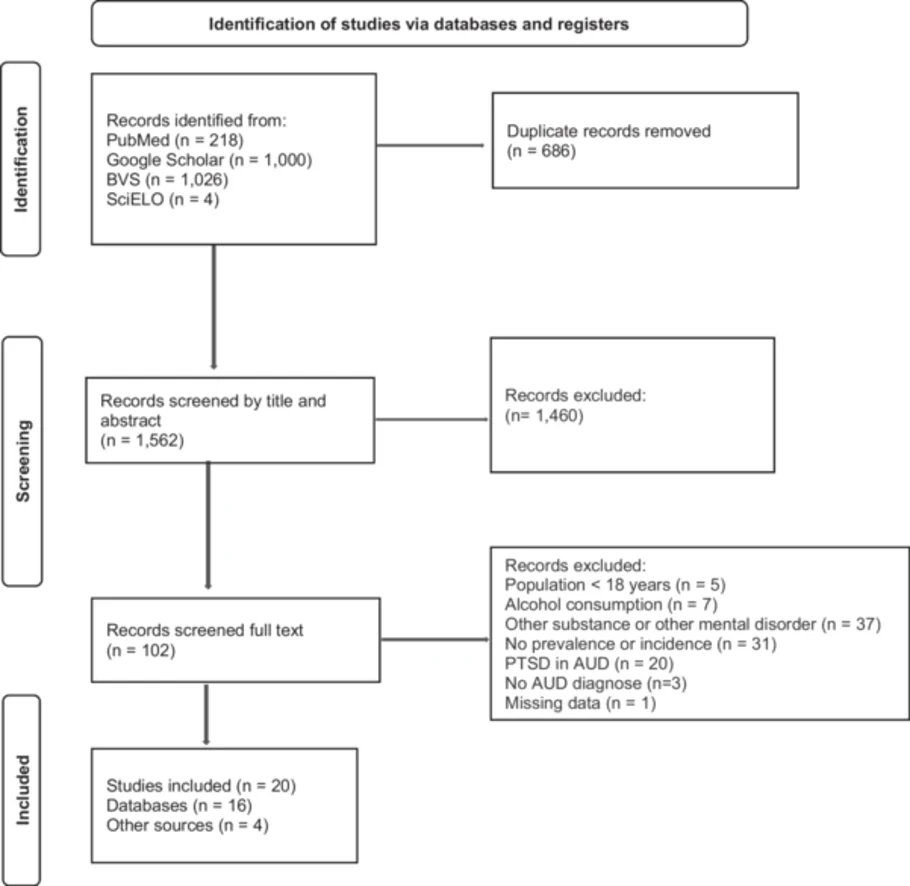
Selection of the studies included in the systematic review. AUD, alcohol use disorder; PTSD, posttraumatic stress disorder.

**Table 1:** Main characteristics of included studies (*N* = 20).

Author, Year	Sample size (Country)	Design/Care setting/Criteria	PTSD assessment	AUD diagnosis and assessment	Prevalence (%)
Blanco et al. 2013	2463 (U.S.) 764 M/1699 W	Cross‐sectional Study/Community/DSM‐IV	AUDADIS‐IV; lifetime	AD; AUDADIS‐IV; lifetime	24.23% *n* = 597
Boudewyns et al. 1991	102 M (U.S.) Veterans	Prospective cohort study/Inpatient/DSM‐III	DIS; non‐lifetime	AUD; DIS; lifetime and current	Current: 32.4% *n* = 33
					Lifetime: 82.4% *n* = 84
Bremner et al. 1996	47 M (U.S.) Veterans	Retrospective cohort study/Inpatient/DSM‐III‐R	SCID; not specified	AD and AA; SCID; lifetime	AD: 78.7% *n* = 37
					AA: 10.6% *n* = 5
Breslau et al. 1997	111 W (U.S.)	Longitudinal community cohort/Community/DSM‐III‐R	NIMH‐DIS; lifetime	AUD; NIMH‐DIS; lifetime	21.6% *n* = 24
Creamer et al. 2001	158 (Australia) 62 M/96 W	Cross‐sectional study/Community/DSM‐IV	CIDI; lifetime	AUD; CIDI; 12‐month	22.15% *n* = 35
Emerson et al. 2017	2368 (U.S.)	Cross‐sectional study/Community/DSM‐5	AUDADIS‐5; lifetime	AUD; AUDADIS‐5; 12‐month	20.69% *n* = 490
Engdahl et al. 1998	140 M (U.S.) Veterans	Cross‐sectional study/Outpatient/DSM‐III‐R	SCID; lifetime	AUD; SCID; lifetime	46.4% *n* = 65
Epstein et al. 2019	270 (U.S.) 175 M/95 W Veterans	Cross‐sectional study/Outpatient/DSM‐IV	SCID; lifetime	AUD; SCID‐IV; lifetime and current	Current: 10% *n* = 27
					Lifetime: 34% *n* = 95
Fox et al. 2020	530 (U.S.) 200 M/330 W	Cross‐sectional study/Community/ICD‐11	AUDADIS‐5; lifetime	AUD; AUDADIS‐5; lifetime	30.1% *n* = 160
Gradus et al. 2017	16,004 (U.S.) 15,044 M/960 W veterans	Cross‐sectional analysis/Outpatient/ICD‐9	ICD‐9; both	AUD; ICD‐9; not specified	15.4% *n* = 2478
Hryvniak and Rosse 1989	311 M (U.S.) Veterans	Retrospective cohort study/Inpatient/DSM‐III‐R	Clinical interview; current	AUD; Clinical interview; current	21.5% *n* = 67
Isabirye et al. 2022	29 (Uganda) 23 M/6 W	Cross‐sectional study/Community/DSM‐5	CAPS‐5; current	AUD; MINI; 12‐month	41.3% *n* = 12
Lewis et al. 2019	160 (U.K.)	Longitudinal cohort study/Community/DSM‐5 (PTSD); DSM‐IV (AUD)	Clinical interview; lifetime	AD; Clinical interview; 12‐month	25.6% *n* = 41
Maguen et al. 2012	159,705 (U.S.) 144,402 M/15,303 W Veterans	Retrospective cohort study/Community/ICD‐9	ICD‐9; lifetime	AUD; ICD‐9; lifetime	27.3% *n* = 43,582
Marmar et al. 2015	33 (U.S.) Veterans	Longitudinal cohort study/Outpatient/DSM‐IV	CAPS‐5; both	AA; SCID; current	3.0% *n* = 1
Muller et al. 2015	140 (Switzerland) 39 M/101 W	Cross‐sectional study/Community/DSM‐IV	SADS‐LA; lifetime	AUD; not reported; lifetime	17.1% *n* = 24
Nadew et al. 2012	121 (Australia)	Cross‐sectional study/Community/ICD‐10/DSM‐IV	CIDI; lifetime	AA; CIDI; lifetime	91% *n* = 111
Pietrzak et al. 2011	2463 (U.S.) 764 M/1699 W	Cross‐sectional study/Community/DSM‐IV	AUDADIS‐IV; lifetime	AUD; AUDADIS‐IV; lifetime	41.8% *n* = 1030
Roszell et al., 1991	48 M (U.S.) Veterans	Cross‐sectional study/Outpatient/DSM‐III‐R	SCID‐III‐R; both	AA and AD; SCID‐III‐R; current and lifetime	Current AA 4.2% (*n* = 2)
					Current AD 33.2% (*n* = 16)
					Lifetime AA 6.3% (*n* = 3)
					Lifetime AD 70.8% (*n* = 34)
Smith et al. 2016	229 (U.S) Veterans	Cross‐sectional study/Outpatient/DSM‐5	AUDADIS‐5; lifetime	AUD; AUDADIS‐5; lifetime	54.51% *n* = 124

Abbreviations: AA, alcohol abuse; AD, alcohol dependence; AUD, alcohol use disorder; AUDADIS, alcohol use disorder and associated disabilities interview schedule; CAPS‐5, clinician‐administered PTSD scale for DSM‐5; CIDI, composite international diagnostic interview; DIS, diagnostic interview schedule; DSM, diagnostic and statistical manual of mental disorders; ICD, international classification of diseases; M, male; MINI, mini international neuropsychiatric interview; NIMH‐DIS, National Institute of Mental Health Diagnostic Interview Schedule; SADS‐LA, Schedule for Affective Disorders and Schizophrenia, Lifetime Version; SCID, structured clinical interview for DSM; W, women/female.

### Participants' characteristics

Most studies included clinical or community‐based samples from the general population (Table 1), with several studies focusing specifically on veterans,^16^, ^17^, ^20^, ^21^ while others examined distinct groups such as police officers,^22^ and Aboriginal^23^ populations. The majority of studies were conducted in North America (*n* = 15), followed by Europe (*n* = 2), two in Oceania,^23^, ^24^ and one study from Africa.^22^ A predominance of studies focused on non‐hospitalized (outpatient or community‐based) populations, whereas only three studies included hospitalized individuals.^16^, ^25^, ^26^ Sample sizes ranged widely, from 29^22^ to 159,705^21^ participants.

### Quality assessment

Quality appraisal with the JBI checklist showed that the methodological quality of the 20 studies was generally high (Table S2). The median study fulfilled 8 of the 9 items, and most used validated diagnostic instruments, applied uniform measurement procedures across participants, and conducted appropriate statistical analyses.

However, limitations were mainly in external‐validity domains: almost half (9/20) did not use an appropriate sample frame, around 45% (9/20) used unclear sample‐size justifications, and approximately 30% (6/20) did not report response rates in a way that allowed formal evaluation. Internal‐validity criteria, such as valid condition identification, standard and reliable measurement, and sufficient coverage of the sample, were met in almost all studies.

### AUDs

A total of 14 studies, including 182,560 participants, investigated the prevalence of AUD among individuals with PTSD. Among these, seven studies^20^, ^21^, ^27^, ^28^, ^29^, ^30^, ^31^ assessed lifetime AUD prevalence only, three assessed AUD over a 12‐month period,^22^, ^24^, ^32^ two evaluated both current and lifetime AUD,^25^, ^33^ and one reported on current AUD only.^26^ One study^34^ did not specify whether the AUD assessment reflected a current or lifetime period; among 16,004 participants with PTSD, 2478 met criteria for AUD, yielding a prevalence of 15.5%. As this study could not be classified by assessment timeframe, it was not included in the quantitative syntheses below.

#### Lifetime AUD prevalence in individuals with PTSD

This multilevel meta‐analysis included 12 studies comprising a total of 45,725 cases of AUD and 166,245 participants with PTSD diagnosis. A multilevel approach was employed because three studies used the same database from NESARC wave 3.^27^, ^30^, ^32^ The pooled estimate of lifetime AUD prevalence among individuals with PTSD was 35.58% (95% CI: 24.48%–48.48%), with substantial heterogeneity (*p* < .0001). Variance decomposition revealed that the entirety of this heterogeneity was attributable to between‐study differences (Level 2 *I* ^2^ = 99.37%, *τ* ^2^ = 0.691), with no variance attributed to the database cluster level (Level 3 *I* ^2^ = 0.0%) (Figure 2).

**Figure 2:**
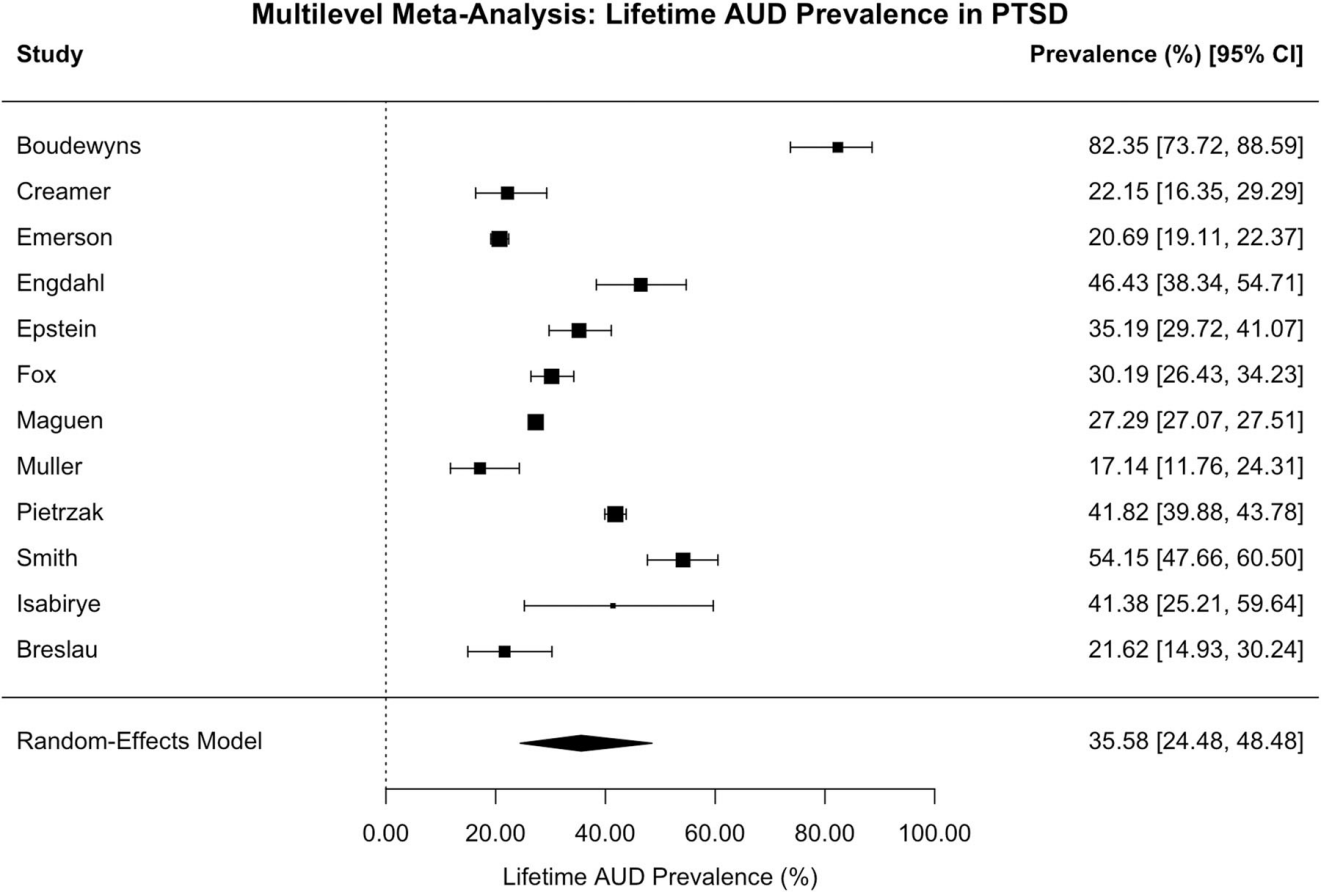
Forest plot of the multilevel meta‐analysis for lifetime prevalence of AUD among individuals with PTSD. AUD, alcohol use disorder; PTSD, posttraumatic stress disorder.

Visual inspection of the funnel plot and Egger's regression test adapted for multilevel models indicated no significant publication bias (*t* = 0.80; *p* = .442), with a bias estimate of 1.98 (SE = 2.48). Consequently, the trim‐and‐fill method did not impute any missing studies, leaving the pooled prevalence estimate unchanged at 35.58% (95% CI: 25.58%–47.02%) (Figure S1). Furthermore, leave‐one‐out sensitivity analyses indicated that no single study disproportionately influenced the overall estimate. The recalculated pooled estimates across iterations ranged from 31.5% to 37.6%, remaining remarkably stable and consistent with the primary result (approximately 36%), underscoring the robustness of the findings despite the consistently high heterogeneity (Figure S2).

Multilevel meta‐regression models were conducted to explore the association between study‐level characteristics and prevalence estimates (Table 2). Each moderator was tested independently due to the limited number of included studies (*k* = 12 for most models; *k* = 11 for the female proportion model). Among the variables assessed, sample type showed a significant association, with community‐based samples reporting lower lifetime AUD prevalence than clinical samples (*β* = –1.230; 95% CI: –1.913 to –0.547; *p* = .002). Care setting (outpatients vs*.* inpatients) showed a significant association, with lower prevalence estimates reported in outpatient samples (*β* = –2.318; 95% CI: –3.699 to –0.937; *p* = .004); however, this result should be interpreted with caution as only one inpatient study^24^ contributed to the inpatient category. Conversely, studies focusing on veterans demonstrated significantly higher AUD prevalence estimates compared to non‐veteran samples (*β* = 1.102; 95% CI: 0.371–1.834; *p* = .007). Furthermore, the proportion of female participants showed a significant inverse association with AUD prevalence (*β* = –1.803; 95% CI: –3.287 to –0.319; *p* = .023). Finally, PTSD diagnostic timeframe (current vs*.* lifetime) was significantly associated with prevalence estimates, with studies requiring a current PTSD diagnosis reporting higher AUD prevalence (*β* = 1.477; 95% CI: 0.266–2.687; *p* = .022); this finding should be interpreted cautiously, as only two studies employed a current PTSD assessment. Other moderators, including decade of publication, geographic region, and diagnostic criteria and AUD assessment timeframe (12‐month vs*.* lifetime/anytime), were not significantly associated with the prevalence estimates.

**Table 2:** Univariable meta‐regression analysis of potential moderators for the lifetime prevalence of AUD among individuals with PTSD.

Covariate	*β*	95% CI	SE	*p*‐value
Decade (per decade)	−0.239	−0.765, 0.287	0.236	.335
Female (proportion)	−1.803	−3.287, −0.319	0.656	.023*
Sample type				
Clinical	(ref)	(ref)	(ref)	(ref)
Community	−1.230	−1.913, −0.547	0.307	.002*
Geographic region				
North America	(ref)	(ref)	(ref)	(ref)
Other	−0.650	−1.897, 0.598	0.560	.273
Diagnostic criteria				
DSM	(ref)	(ref)	(ref)	(ref)
ICD	−0.090	−1.387, 1.207	0.582	.881
Care setting				
Inpatients	(ref)	(ref)	(ref)	(ref)
Outpatients	−2.318	−3.699, −0.937	0.620	.004*
Veteran status				
Non‐veteran	(ref)	(ref)	(ref)	(ref)
Veteran	1.102	0.371, 1.834	0.328	.007*
AUD time frame				
12 months	(ref)	(ref)	(ref)	(ref)
Lifetime	−0.310	−1.507, 0.887	0.537	.577
PTSD time frame				
Lifetime	(ref)	(ref)	(ref)	(ref)
Current	1.477	0.266, 2.687	0.543	.022*

*Note*: Estimates from multilevel meta‐regression (random intercepts at the study and database levels; *k* = 12, except Female where *k* = 11 due to one study with a missing value). Care Setting includes a single inpatient study (Boudewyns), so the outpatients estimate should be interpreted with caution.

Abbreviations: *β*, regression coefficient (logit scale); AUD, alcohol use disorder; CI, confidence interval; DSM, diagnostic and statistical manual of mental disorders; ICD, international classification of diseases; PTSD, posttraumatic stress disorder; ref, reference category; SE, standard error.

* Statistical significance at *p* < .05.

#### Current AUD prevalence in individuals with PTSD

Three studies^25^, ^26^, ^33^ reported point‐prevalence estimates of AUD among individuals with PTSD, comprising a total of 116 cases across 683 participants. All three were conducted in the United States in clinical veteran samples. Reported prevalence rates ranged from 10.0% in Epstein et al.^33^ to 32.4% in Boudewyns et al.,^25^ with Hryvniak and Rosse^26^ reporting an intermediate estimate of 21.5%. Assessment methods varied across studies in both AUD timeframe definition and PTSD diagnostic criteria; details are provided in Table 1. Given the limited number of eligible studies, the absence of community‐based or non‐veteran samples, and the heterogeneity in both AUD and PTSD assessment methods, formal meta‐analysis was not conducted; findings are presented descriptively and should be interpreted accordingly.

### Alcohol dependence (AD)

Four studies^16^, ^35^, ^36^, ^37^ examined AD among individuals with PTSD, encompassing a total of 2718 participants, of whom 709 met diagnostic criteria for AD at a lifetime assessment. Only two^16^, ^36^ studies included veteran samples. Given the limited number of independent studies, we did not perform a meta‐analysis or meta‐regression for this outcome. Additionally, it is worth noting that although Blanco et al.^37^ utilized the same sample as Pietrzak et al.^29^ (NESARC II database), it was retained in our descriptive analysis because it provides a specific subclassification for alcohol dependence.

### Alcohol abuse (AA)

A total of four studies^16^, ^17^, ^23^, ^36^ investigated AA in individuals with PTSD, comprising a combined sample of 249 participants, of whom 120 met the diagnostic criteria for AA. Three studies used DSM criteria, while one employed a mixed ICD‐10/DSM‐IV approach.^23^ Three studies focused on veteran populations, while only one included a broader sample. Notably, two records derived from the same study^36^ reported both lifetime and current AA outcomes. Formal meta‐analysis was not conducted given the small number of independent studies.

## DISCUSSION

The pooled lifetime prevalence of AUD among individuals with PTSD was approximately 36% (95% CI: 24.48%–48.48%), indicating that more than one in three people with PTSD have experienced AUD at some point. Despite overall consistency in findings, heterogeneity across studies was consistently high (*I* ^2^ = 99.37%), reflecting wide variability in comorbidity rates depending on sample characteristics. Meta‐regression analyses provided important insights into this variation. Outpatient samples showed significantly lower lifetime AUD prevalence than inpatient settings; however, only one study (Boudewyns et al.^25^) contributed to the inpatient category, so this estimate warrants caution. All three inpatient studies in the review enrolled combat veterans with chronic PTSD in psychiatric hospital settings,^16^, ^25^, ^26^ where referral patterns concentrate more severe and comorbid cases. Beyond care setting, sample source (clinical vs*.* community) also emerged as a significant moderator (*β* = –1.230; *p* = .002), with community‐based studies reporting lower lifetime AUD prevalence than clinical samples. This pattern is consistent with treatment‐seeking biases: individuals presenting to clinical settings tend to exhibit greater illness severity and comorbidity burden than those identified through community surveys, which recruit across the full spectrum of symptom severity.^19^ Notably, samples focusing on veterans^20^, ^21^, ^25^, ^30^, ^33^ demonstrated significantly higher lifetime AUD prevalence compared to civilian cohorts, reflecting the multifactorial vulnerability of this population, including elevated occupational trauma exposure,^38^ and high rates of comorbid traumatic brain injury and chronic pain.^18^

Importantly, veteran status marks a high‐risk context rather than a direct index of combat exposure;^16^, ^17^ the elevated AUD prevalence in these samples likely reflects converging influences beyond trauma itself, including permissive military drinking cultures,^39^ occupational and deployment‐related stressors,^18^ barriers to timely mental‐health care,^40^ and the predominance of men in veteran cohorts.^7^, ^8^ Attributing it solely to combat exposure would overlook these structural and cultural contributors. Additionally, meta‐regression revealed that a higher proportion of female participants was significantly associated with lower lifetime AUD prevalence, aligning with broader epidemiological trends that consistently indicate higher rates of AUDs among men.^7^, ^8^ However, this finding should be interpreted in light of evidence documenting a progressive narrowing of the sex gap in AUD over recent decades, with prevalence rising more steeply among women across multiple high‐income countries.^7^, ^8^ The persistence of a significant female proportion coefficient in our meta‐regression may therefore partially reflect the older diagnostic era of several included studies, and future reviews incorporating more recent data may observe an attenuated or absent sex‐based moderating effect. Furthermore, the timeframe of the PTSD diagnosis emerged as a significant moderator (*β* = 1.477; *p* = .022), with studies requiring a current active PTSD diagnosis reporting higher comorbid AUD prevalence than those accepting a lifetime diagnosis. This may reflect greater clinical severity in samples with ongoing PTSD; however, given that only two studies^22^, ^25^ employed a current PTSD assessment, this finding warrants cautious interpretation and replication.

Moreover, concerns regarding the evolving diagnostic criteria across different DSM editions (e.g., from DSM‐III‐R/IV to DSM‐5) were addressed through our meta‐regression analysis. We found that the decade of publication, which serves as a proxy for the diagnostic era, was not significantly associated with prevalence estimates. This suggests that the core construct of PTSD and its high comorbidity with AUD remain robust and detectable across nosological iterations, justifying the aggregation of these studies. Importantly, studies conducted prior to DSM‐5 assessed alcohol abuse and alcohol dependence as separate diagnostic categories; for the purposes of this review, both were operationally treated as indicators of the AUD spectrum, consistent with the DSM‐5 reconceptualization.^1^ The non‐significant decade effect supports this analytic decision.

Our findings align with a longstanding body of literature documenting the strong association between PTSD and substance use, particularly alcohol. Data from the NESARC‐III indicated that 25.19% of individuals with PTSD had past‐year AUD.^41^ Notably, a separate NESARC‐III analysis that restricted its sample to cases where PTSD onset demonstrably preceded AUD was excluded from our meta‐analysis because it examined a specific causal sequence rather than the total comorbidity burden; the fact that similarly high rates were observed in that restricted sample further supports the robustness of the PTSD–AUD association across methodological approaches.^41^ Similarly, data from the Australian National Survey of Mental Health and Well‐being estimated that approximately 37% of men with PTSD met criteria for past‐year AUD, compared to 12% of women,^24^ a gender disparity directly supported by our meta‐regression results. Together, these studies suggest that the PTSD–AUD link is robust across clinical samples, veteran cohorts, and general population surveys.

While an extensive body of research has historically examined PTSD emergence within AUD populations,^38^, ^42^ the two literatures converge: the same review reporting alcohol misuse in 9.8%–61.3% of individuals with PTSD found PTSD in 2.0%–63.0% of those with alcohol misuse,^15^ and our pooled estimate of 35.58% falls within both ranges. The present review nonetheless advances the literature by systematically quantifying the burden of AUD specifically among individuals already diagnosed with PTSD, a population less frequently targeted in meta‐analytic syntheses. Beyond confirming the high overall prevalence, our analysis clarifies sources of variability: AUD prevalence is significantly moderated by care setting and veteran status. Sampling on PTSD rather than on AUD treatment, and including community alongside clinical samples, avoids the referral filter of treatment‐seeking cohorts,^19^ a choice validated by the significant effect of sample source in our meta‐regression.

The strong overlap between PTSD and AUD is supported by convergent behavioral and neurobiological evidence. As outlined in the Introduction, behavioral models, self‐medication^11^ and negative reinforcement/affective processing,^12^ account for how alcohol use becomes entrenched in PTSD; here we focus on the neurobiological substrates most relevant to this comorbidity. At the neurobiological level, this comorbidity is underpinned by shared biological substrates. Dysregulation of the hypothalamic‐pituitary‐adrenal axis, sensitization of corticotropin‐releasing factor systems, and disrupted mesolimbic reward circuitry, including the ventral tegmental area and nucleus accumbens, are implicated in both PTSD and AUD, providing a biological scaffolding for their mutual reinforcement.^13^ At the genetic level, this specificity is informative: rather than a general predisposition toward drinking, the shared liability between PTSD and AUD reflects an overlapping genetic architecture for psychopathology and disorder‐level, harmful alcohol use.^14^

Translating these findings into clinical practice, the high and context‐dependent prevalence of AUD among individuals with PTSD has clear implications for both screening and treatment. The substantially elevated AUD prevalence in inpatient and veteran populations supports differential allocation of screening resources: systematic, routine AUD screening should be prioritized in VA settings, specialized PTSD inpatient programs, and psychiatric acute care units.^25^, ^26^, ^38^ In these high‐prevalence contexts, clinical assessment should address alcohol use proactively as part of standard PTSD evaluation.^42^, ^43^

High AUD prevalence also underscores the inadequacy of treating PTSD in isolation. Because alcohol frequently serves as a compensatory coping mechanism, addressing trauma without simultaneously managing alcohol use can precipitate symptom rebound, compromising treatment engagement and outcomes for both conditions.^42^ Evidence‐based integrated psychotherapeutic approaches exist for this comorbidity: Concurrent Treatment of PTSD and Substance Use Disorders Using Prolonged Exposure (COPE)^44^ combines trauma‐focused prolonged exposure with cognitive‐behavioral strategies for substance use; Seeking Safety^45^ provides present‐focused, integrated skills training for trauma and substance use without requiring trauma processing; and adaptations of cognitive processing therapy (CPT) have also been studied in comorbid populations.^46^, ^47^ Pharmacologically, evidence‐based treatments for each condition can be used within integrated care frameworks. Naltrexone, FDA‐approved for AUD, has demonstrated efficacy in reducing alcohol consumption in individuals with comorbid PTSD and AUD and, importantly, does not exacerbate PTSD symptoms when combined with prolonged exposure therapy.^42^, ^48^ SSRIs (sertraline and paroxetine), the only FDA‐approved pharmacotherapies for PTSD, may provide additional benefits in this population, though their specific effects on comorbid AUD remain modest.^42^ These approaches should be prioritized particularly for the veteran and inpatient populations identified in this review as carrying the greatest burden.

## LIMITATIONS

As with any research synthesis, our meta‐analysis is not without limitations. Although we included 20 studies in total, the evidence base for certain subgroups was small. For example, only four studies examined alcohol dependence and four examined alcohol abuse separately in individuals with PTSD. This scarcity precluded the execution of separate meta‐analyses or meta‐regressions for these specific categories, compelling us to focus instead on combined AUD measures, which may have blurred meaningful diagnostic differences. Furthermore, the limited number of studies available for our primary outcome (*k* = 12 for lifetime AUD; formal meta‐analysis could not be conducted for current AUD due to an insufficient number of eligible studies) restricted our meta‐regression analyses to univariable models. This constrained our ability to simultaneously adjust for multiple covariates or fully explore the complex interactions driving the high heterogeneity we observed (*I* ^2^ = 99.4%). Relatedly, age could not be evaluated as a moderator because it was inconsistently reported across studies (typically as means only, without measures of dispersion), precluding a valid meta‐regression.

Importantly, the literature is geographically skewed toward high‐income, Western settings, with limited data from low‐ and middle‐income countries and other underrepresented regions; consequently, generalizability across cultural norms, alcohol availability, and trauma exposures remains uncertain.

Regarding diagnostic assessment, while all included studies utilized DSM or ICD‐based definitions, assessment methods ranged from structured clinical interviews to administrative diagnostic codes (e.g., ICD‐9), the latter of which may capture prevalent rather than incident cases and are subject to coding inaccuracies. Consequently, heterogeneity in the precision of case ascertainment across studies may influence the specificity of our prevalence estimates.

A further limitation is the predominance of cross‐sectional designs, which precludes assessment of temporal sequences between PTSD onset and AUD development. Lifetime AUD prevalence estimates reflect cumulative diagnostic burden regardless of participants' current clinical status; however, point‐prevalence estimates of current AUD, presented here only descriptively, are more sensitive to the clinical state at the time of assessment and should be interpreted accordingly. Representativeness is another concern. Many studies drew on clinical samples, such as veterans in treatment or hospital patients, who may not reflect the experiences of the broader population. Indeed, our meta‐regression confirmed that veteran status and care setting (inpatients) significantly moderated the prevalence estimates. Consequently, the pooled rates may be somewhat inflated by the overrepresentation of these highly burdened, specialized groups.

Finally, while our analysis did not detect significant variance based on the decade of publication, the pooling of studies across different diagnostic eras (DSM‐III, DSM‐IV, and DSM‐5) introduces inherent heterogeneity. Changes in PTSD symptom clusters and the consolidation of alcohol abuse and dependence into a single AUD spectrum in DSM‐5 may influence case identification in ways that aggregate statistics cannot fully capture.

## CONCLUSION

This review makes a distinct contribution by adopting a PTSD‐focused perspective, systematically quantifying the burden of AUD within samples defined by either a current or lifetime PTSD diagnosis, rather than the more common approach of detecting PTSD within AUD treatment populations. This framing reflects a clinically relevant assessment sequence and does not imply that PTSD preceded AUD onset. Beyond confirming a high overall prevalence (~36% lifetime), the meta‐regression identified specific, actionable moderators: inpatient care settings, clinical sample sources, and veteran status were associated with substantially higher AUD prevalence, whereas a higher proportion of female participants was inversely associated with lifetime rates. These findings move beyond generic calls for integrated care by specifying the populations and contexts in which that burden is greatest. Clinically, this evidence supports the differential allocation of screening resources and treatment intensity, particularly for veteran and inpatient populations. Prospective and longitudinal designs are needed to establish temporal pathways and test mechanistic hypotheses. Future research should also prioritize low‐ and middle‐income settings and populations underrepresented in the current evidence base.

## CONFLICT OF INTEREST STATEMENT

The authors declare no conflicts of interest.

## Supporting information

Supporting File 1.

Supporting File 2.

Supporting File 3.

Supporting File 4.

Supporting File 5.

## Data Availability

Data sharing is not applicable to this article as no datasets were generated or analyzed during the current study.
