# Supplementary material for: How common is alcohol use disorder in posttraumatic stress disorder? A systematic review, meta‐analysis, and moderator analysis
Source: Am J Addict. 2026 Sep 16;35(6):727–38. doi: 10.1111/ajad.70200 (PMC13712570; doi:10.1111/ajad.70200)
Supplement: Supplementary file 2 [file AJAD-35-727-s004.docx]

Table S2. Results of the Quality Assessment.

| **Author** | Q1 | Q2 | Q3 | Q4 | Q5 | Q6 | Q7 | Q8 | Q9 | |
| --- | --- | --- | --- | --- | --- | --- | --- | --- | --- | --- |
| Blanco et al. (2013) | 1 | 1 | 1 | 1 | 1 | 1 | 1 | 1 | 1 | |
| Boudewyns et al. (1991) | 2 | 1 | 3 | 1 | 1 | 1 | 1 | 1 | 1 | |
| Bremner et al. (1996) | 2 | 1 | 3 | 1 | 1 | 1 | 1 | 1 | 1 | |
| Breslau et al. (1997) | 2 | 1 | 1 | 1 | 1 | 1 | 1 | 1 | 1 | |
| Creamer et al. (2001) | 1 | 1 | 1 | 1 | 1 | 1 | 1 | 1 | 1 | |
| Emerson et al. (2017) | 1 | 1 | 1 | 1 | 1 | 1 | 1 | 1 | 1 | |
| Engdahl et al. (1998) | 2 | 1 | 3 | 1 | 1 | 1 | 1 | 1 | 1 | |
| Epstein et al. (2019) | 2 | 2 | 1 | 1 | 1 | 1 | 1 | 1 | 3 | |
| Fox et al. (2020) | 1 | 1 | 2 | 1 | 1 | 1 | 1 | 1 | 1 | |
| Gradus et al. (2017) | 2 | 1 | 1 | 1 | 1 | 1 | 1 | 1 | 4 | |
| Hryvniak & Rosse (1989) | 2 | 1 | 2 | 1 | 1 | 1 | 1 | 1 | 4 | |
| Isabirye et al. (2022) | 1 | 1 | 3 | 1 | 1 | 1 | 1 | 1 | 1 | |
| Lewis et al. (2019) | 1 | 1 | 1 | 1 | 1 | 1 | 1 | 1 | 1 | |
| Maguen et al. (2012) | 1 | 1 | 1 | 1 | 1 | 1 | 1 | 1 | 4 | |
| Marmar et al. (2015) | 1 | 1 | 1 | 1 | 1 | 1 | 1 | 1 | 1 | |
| Müller et al. (2015) | 1 | 1 | 3 | 1 | 1 | 1 | 1 | 1 | 1 | |
| Nadew (2012) | 2 | 1 | 3 | 1 | 1 | 1 | 1 | 1 | 3 | |
| Pietrzak et al. (2011) | 1 | 1 | 1 | 1 | 1 | 1 | 1 | 1 | 1 | |
| Roszell et al. (1991) | 2 | 1 | 3 | 1 | 1 | 1 | 1 | 1 | 4 | |
| Smith et al. (2016) | 1 | 1 | 1 | 1 | 1 | 1 | 1 | 1 | 1 | |
| 1 = yes 2 = no 3 = unclear 4 = not applicable | | | | | | | | | | |
| ***JBI Critical Appraisal Checklist for studies reporting prevalence data***   1. Was the sample frame appropriate to address the target population? 2. Were study participants sampled in an appropriate way? 3. Was the sample size adequate? 4. Were the study subjects and the setting described in detail? 5. Was the data analysis conducted with sufficient coverage of the identified sample? 6. Were valid methods used for the identification of the condition? 7. Was the condition measured in a standard, reliable way for all participants? 8. Was there appropriate statistical analysis? 9. Was the response rate adequate, and if not, was the low response rate managed appropriately? | | | | | | | | | |  |
