# Supplementary material for: How common is alcohol use disorder in posttraumatic stress disorder? A systematic review, meta‐analysis, and moderator analysis
Source: Am J Addict. 2026 Sep 16;35(6):727–38. doi: 10.1111/ajad.70200 (PMC13712570; doi:10.1111/ajad.70200)
Supplement: Supplementary file 3 [file AJAD-35-727-s005.docx]

## Table S1. Search strategy

| Database | Search | Terms | Results |
| --- | --- | --- | --- |
| Pubmed | 1 | “prevalence”[Mesh] OR “incidence”[Mesh] OR “epidemiology”[Mesh] | 711,920 |
| Pubmed | 2 | ("alcohol dependence"[tiab] OR "alcohol-related disorders"[Mesh] OR "alcohol abuse"[tiab]) | 139,572 |
| Pubmed | 3 | "Posttraumatic Stress Disorder"[tiab] OR "Stress Disorders, Post-Traumatic"[Mesh] OR "Stress Disorders, Traumatic"[Mesh] | 59,791 |
| Pubmed | 4 | 1 AND 2 AND 3 | 218 |
| Google Scholar | 1 | ("prevalence" OR "incidence" OR "epidemiology") | 769,000 |
| Google Scholar | 2 | ("alcohol dependence" OR "alcohol-related disorders" OR "alcohol abuse") | 337,000 |
| Google Scholar | 3 | ("Posttraumatic Stress Disorder" OR "post-traumatic stress disorder" OR "traumatic stress disorder" OR PTSD) | 265,000 |
| Google Scholar | 4 | 1 AND 2 AND 3 | 18,400 |
| Google Scholar | 5 | Availability to screening on Google Scholar | 1,000 |
| BVS | 1 | “prevalence” OR “incidence” OR “epidemiology” | 636,968 |
| BVS | 2 | ("alcohol dependence" OR "alcohol-related disorders" OR "alcohol abuse") | 157,916 |
| BVS | 3 | ("post-traumatic stress disorder" OR "traumatic stress disorder" OR PTSD) | 57,530 |
| BVS | 4 | (English) 2 and 3 | 1,642 |
| BVS | 5 | (Portuguese) 2 and 3 | 952 |
| BVS | 6 | (Spanish) 2 and 3 | 953 |
| BVS | 7 | 1 AND 2 AND 4 | 1,026 |
| SciElo | 1 | (Spanish) “prevalence” OR “incidence”OR “epidemiologí" | 78,991 |
| SciElo | 2 | (Português) ("prevalência" OR "incidência" OR "epidemiologia") | 78,991 |
| SciElo | 3 | (English) (“prevalence” OR “incidence”OR “epidemiology”) | 83,894 |
| SciElo | 4 | (Spanish) ("dependencia del alcohol" OR "trastornos relacionados con el alcohol" OR "consumo nocivo de alcohol" | 68 |
| SciElo | 5 | (Português) ("dependência de álcool" OR "transtornos relacionados ao álcool" OR "uso nocivo de álcool") | 100 |
| SciElo | 6 | (English) ("alcohol dependence" OR "alcohol-related disorders" OR "alcohol abuse") | 798 |
| SciElo | 7 | ("post-traumatic stress disorder" OR "traumatic stress disorder" OR PTSD) | 650 |
| SciElo | 8 | 3 AND 6 AND 7 | 7 |
